# Standardised Neuropsychological Assessment for the Selection of Patients Undergoing DBS for Parkinson's Disease

**DOI:** 10.1155/2018/4328371

**Published:** 2018-06-03

**Authors:** Jennifer A. Foley, Tom Foltynie, Patricia Limousin, Lisa Cipolotti

**Affiliations:** ^1^National Hospital for Neurology and Neurosurgery, Queen Square, London, UK; ^2^UCL Institute of Neurology, Queen Square, London, UK; ^3^Dipartimento di Scienze Psicologiche, Pedagogiche e della Formazione, Università degli Studi di Palermo, Palermo, Italy

## Abstract

DBS is an increasingly offered advanced treatment for Parkinson's disease (PD). Neuropsychological assessment is considered to be an important part of the screening for selection of candidates for this treatment. However, no standardised screening procedure currently exists. In this study, we examined the use of our standardised neuropsychological assessment for the evaluation of surgical candidates and to identify risk factors for subsequent decline in cognition and mood. A total of 40 patients were assessed before and after DBS. Evaluation of mood and case notes review was also undertaken. Before DBS, patients with PD demonstrated frequent impairments in intellectual functioning, memory, attention, and executive function, as well as high rates of mood disorder. Post-DBS, there was a general decline in verbal fluency only, and in one patient, we documented an immediate and irreversible global cognitive decline, which was associated with older age and more encompassing cognitive deficits at baseline. Case note review revealed that a high proportion of patients developed mood disorder, which was associated with higher levels of depression at baseline and greater reduction in levodopa medication. We conclude that our neuropsychological assessment is suitable for the screening of candidates and can identify baseline risk factors, which requires careful consideration before and after surgery.

## 1. Introduction

Drug therapies for advanced Parkinson's disease (PD) can be unsatisfactory, with unwanted side effects, and/or insufficient control of disabling motor symptoms. Thus, there has been resurgence in interest in surgical treatments, with deep brain stimulation (DBS) now increasingly offered as an option. DBS is the chronic, high-frequency electrical stimulation of most usually the subthalamic nucleus (STN) or internal segment of the globus pallidus (GPi) [1], which is thought to alter the pattern of neural activity, with resulting beneficial effects upon motor function [2]. Its success relies heavily upon appropriate selection of candidates, which in turn relies in part upon neuropsychological screening [3, 4]. However, no standardised screening procedure currently exists, and it remains unclear what level of cognitive dysfunction precludes successful surgery. In this study, we discuss the limitations of existing presurgical protocols and evaluate the use of standardised neuropsychological assessment in a sample of patients undergoing DBS for PD. We describe patients' performance on this neuropsychological assessment before and after DBS and identify potential baseline predictors of after DBS decline, which warrant further investigation.

DBS has been shown to be relatively safe, with few negative events occurring during or following surgery, when performed on appropriate candidates [5]. However, STN DBS is thought to result in better improvements in motor control; there is some evidence to suggest that it also poses a greater risk of negatively affecting speech articulation, impulsive behaviours, and/or mood [6–10], and therefore, GPi DBS may be preferred for patients presenting with these difficulties. However, marked global cognitive deterioration has also been reported [11, 12], and mild decline in verbal fluency has frequently been documented [13–30].

Decline in cognitive functioning following DBS has been found to be more common in patients who are older, especially above 70 years [29, 31], particularly affecting frontal executive functions [28]. Yet others have cautioned against a strict age criterion, as many people older than 70 can demonstrate good outcomes [32, 33]. Indeed, it has been reported that other factors, particularly cognitive performance, may be more useful as predictors of postoperative decline [3, 29, 34, 35]. Several studies have suggested that lower cognitive functioning at baseline is predictive of poorer cognitive outcome following surgery [36], perhaps because of lower “cognitive reserve” [35]. It has even been suggested that the presence of any cognitive deficits at baseline, particularly in executive function and memory, should serve as exclusion [36, 37]. However, PD is usually accompanied by at least mild cognitive deficits, particularly in executive function, and the evolution of a dementia is rather insidious, without any clear boundary features. Thus, it remains unclear what level of impairment should constitute a contraindication to surgery.

Despite the widespread agreement on the importance of appropriate screening and careful selection of surgery candidates, to the best of our knowledge no standardised neuropsychological assessment procedure currently exists. The Consensus on DBS for PD [38] published guidance on presurgical screening and selection of patients but did not provide a presurgical neuropsychological protocol. Rather, they listed an extensive range of neuropsychological domains to be assessed and tests commonly used. The tests listed ranged from very brief screens (including the MMSE) to very long and extensive batteries (such as the Wechsler Memory Scales and Delis–Kaplan Executive Functioning System). They stated that tests chosen should be reliable and valid, with adequate normative data for referencing performance. However, without any guidance on how to choose between the vast range of tests, nor how to interpret scores when deciding on suitability for surgery, it remains unclear how to use neuropsychological assessments to identify candidates suitable for DBS. Indeed, defining what constitutes unacceptable cognitive dysfunction remains the most controversial aspect of patient selection [2].

Moreover, there is scant official guidance. The British Psychological Society [39] recommends that candidates undergo presurgery neuropsychological evaluation but does not describe what this should consist of. The Australian guidelines [40] simply recommend that patients should be able to give a good account of themselves and capable of giving informed consent. Of course, even marked cognitive impairment may be masked by higher levels of cognitive reserve and/or fluctuating levels of attention and vice versa; gross physical and speech disability may mask intact cognition. Thus, the absence of any firm guidelines for the assessment and interpretation of cognitive performance clearly poses a significant hurdle for the appropriate selection of candidates for DBS.

In lieu of such guidance, several studies have relied upon brief cognitive screens only, such as the Mini-Mental State Examination (MMSE) [41]. This may be criticised for a number of reasons. Firstly, the MMSE comprises very few subtests that are sensitive to the typical cognitive dysfunction displayed in PD, namely, executive dysfunction [42–48] and cognitive slowing [49–52] and thus is insufficient for detecting cognitive impairment [53, 54]. Secondly, the MMSE suffers from significant ceiling and floor effects [55], so cannot capture very mild nor very severe cognitive dysfunction. Thirdly, MMSE scores are affected by age and education [56, 57], so that a low score in an older person with minimal formal schooling may present a false positive for dementia. Fourthly, an individual may attain a low MMSE score for a number of noncognitive reasons, including poor speech intelligibility, high levels of anxiety, fatigue, and distracting dyskinesia. Therefore, a low score on this test should not necessarily be used to preclude surgery.

Moreover, such brief screening tools do not permit scrutiny of the wider cognitive profile, important for confirming diagnosis. Many cases of “failed” DBS have been later found to have atypical Parkinsonian syndromes, known not to benefit from DBS [58]. Thus, there is a need to identify a suitable presurgical neuropsychological protocol, which is sufficiently sensitive to both the typical cognitive dysfunction displayed in PD and atypical cognitive decline, as seen in other Parkinsonian disorders and has clear guidelines for its interpretation.

In addition to changes in cognition, there are also a few reports of dramatic deterioration in mood and greatly increased apathy following DBS [22, 59–61], with an associated elevated risk of suicide despite successful reduction of motor symptoms [62, 63]. As such deterioration can clearly negate any potential benefits [64], it is essential that candidates at high risk of such postoperative decline are identified at baseline. Specifically, postoperative risk of suicide has been associated with higher levels of mood disorder, apathy, and/or family or social stress at baseline [65, 66]. This may not only reflect the additional stressor of surgery [67, 68] but also the direct effects of the stimulation itself [69] and any reductions in dopaminergic medication [70, 71]. As mood disorder is so prevalent in PD and may reduce with improvements in motor symptoms following surgery [35], it remains unclear what level of mood disorder should act as an absolute contraindication for DBS.

Thus, the aims of this study were to evaluate the use of our standardised neuropsychological protocol in the evaluation of patients undergoing DBS for PD in order to identify any contraindication for surgery and to be sensitive to changes following DBS.

## 2. Methods

### 2.1. Participants

A total of 40 patients (29 male, 11 female) who underwent DBS took part in this study. All patients had had a diagnosis of idiopathic PD for at least five years (according to Queen Square Brain Bank criteria), were younger than 70 years, and suffered from disabling motor complications despite optimal treatment. Each patient underwent multidisciplinary evaluation to decide on suitability for DBS. Formal levodopa challenge confirmed dopaminergic drug responsiveness. A structural MRI was obtained to exclude surgical contraindications, such as advanced brain atrophy, white matter changes, or any other abnormality contraindicating surgery. Detailed neuropsychological and neuropsychiatric assessments excluded patients with significant cognitive impairment and/or psychiatric comorbidities. Contraindications for STN DBS included the presence of clinically relevant speech difficulties and cognitive impairment. The final decision regarding suitability for DBS and appropriate target for each patient was taken during a joint meeting of patient, immediate family, neurologist(s), and neurosurgeon(s).

Motor status was evaluated using part III of the Unified Parkinson's Disease Rating Scale (UPDRS-III). Prior to surgery, patients were assessed in the practically defined “off state” after overnight withdrawal of anti-Parkinsonian drugs and the “on state,” following a levodopa challenge using a suprathreshold dose of oral levodopa. After DBS, motor assessments were sequentially performed under the following conditions, in open fashion: off medication/on stimulation (with stimulation switched on after 12 h medication withdrawal) and on medication/on stimulation (1 h after the administration of a routine dose of levodopa while stimulation was reintroduced). All medications before and after surgery were recorded, noting any dopamine agonist treatment, and levodopa-equivalent dosage was calculated (www.parkinsonsmeasurement.org). History of impulse control disorder was recorded by reviewing the medical notes and noting any mention of compulsive gambling, eating, shopping, or sexual behaviour before or after DBS.

All patients underwent assessment of neuropsychological and mood functioning before and after surgery, under optimal conditions. Thus, preoperatively, this was in the on medication and postoperatively on stimulation/on medication. The postoperative assessment was performed a mean of 19.60 months after surgery (range = 1–54; SD = 11.56). This broad range reflected the early recall of one patient following concern about cognition immediately following DBS, as well as later routine follow-up of cognitively intact patients after surgery.

The most appropriate DBS target was chosen on clinical grounds based on patient motor phenotype, imaging, and preoperative cognitive assessment. Twenty-eight patients underwent bilateral STN DBS and twelve bilateral GPi DBS.

### 2.2. Neuropsychological Assessment

The tests included general screening and IQ measures, as well as tests of specific cognitive functions. This was to enable both the quantification of any intellectual deficit and the elucidation of specific cognitive profiles. Thus, the measures included tests of general cognitive functioning, memory, language, visuoperceptual ability, attention, executive functions, and speed of processing. The tests chosen were considered to have acceptable test validity and reliability, as described below. The assessments took around two hours to complete and were as follows:

1. The MMSE was used as a screening test of global cognitive functioning [41]. It is not sufficient as a measure of cognition in Parkinson's disease [53], but as the “gold standard” screening instrument, it permits easy comparison between studies.
2. Vocabulary, similarities, arithmetic, and digit span subtest scores from the Wechsler Adult Intelligence Scale-Third Edition (WAIS-III) [72] were prorated to generate verbal IQ (VIQ). Picture Completion and Matrix Reasoning subtest scores were prorated to generate scores for nonverbal IQ (PIQ). The WAIS-III has been found to have good sensitivity and specificity for cognitive disorders [73] and good reliability for Parkinson's disease [74].
3. The National Adult Reading Test-Revised (NART-R) [75] was used to estimate the premorbid level of intellectual functioning, by generating each patient's Predicted Full-Scale IQ (PFSIQ). The NART-R has very good interrater and test-retest reliability, and good validity, although suffers from a ceiling effect limiting prediction of IQ scores beyond 125.
4. Memory was assessed using the following:
  - The Warrington Words and Faces Recognition Memory Tests (RMTs) [76] were used to assess recognition memory. The RMT correlates well with other measures of memory and has adequate reliability for patients with neurological disorders [77, 78].
  - The People and Shapes subtests from the Doors and People Test were used to assess verbal and visual recall memory (D&P) [79]. These tests have sufficient validity and reliability [80] and are recommended for assessing recall in PD [81].
5. The Graded Naming Test (GNT) [82] was used to assess language. The GNT has good test-retest reliability and is well suited for detecting any gradual changes in performance over time [83]. Moreover, it is sensitive to cognitive impairment in Parkinson's disease [84].
6. The Silhouettes subtest from the Visual Object and Space Perception Battery (VOSP) [85] was used to assess visuoperceptual functioning. This test has been validated as a test of object perception [86] and is sensitive to visuospatial impairment seen in PD dementia [87] and atypical PD [88].
7. Elevator Counting and Elevator Counting with Distraction subtests from the Test of Everyday Attention (TEA) [89] were used to assess sustained and selective attention. These tests have high test-retest reliability and correlate with other measures of attention. Furthermore, these tests have been shown to be sensitive to Parkinsonian disorders, including Lewy body dementia [90].
8. Executive functioning was assessed using the following:
  - FAS and Category subtests from the Delis–Kaplan Executive Function System (DKEFS) [91] were used to assess verbal fluency. The tests have been standardised and found to be sufficiently reliable [92].
  - The Stroop [93] was used to assess verbal inhibition. It has high reliability [94] and is sensitive to cognitive deficits in PD [95].
  - The Hayling and Brixton tests [96] were used to assess verbal suppression/strategy formation [97] and nonverbal set-shifting, respectively. They have moderate sensitivity and specificity for detecting executive dysfunction [98] and are sensitive to PD [99, 100].
9. The Symbol Search and Digit Symbol Coding subtests from the WAIS-III [72] were used to assess processing speed. These tests have been shown to be sensitive to PD [101].

### 2.3. Mood Assessment

All patients were screened for mood disorder using the Hospital Anxiety and Depression Scale (HADS) [102] and the Apathy Evaluation Scale (AES) [103]. These tests have been validated for use in PD [104, 105].

### 2.4. Case Note Review

The case notes were reviewed by one clinical neuropsychologist (JAF) to identify any change in cognition, mood, or behaviour since DBS, as highlighted by the surgery team, neurologists, or nursing staff. Any mention of decline in memory, attention, perception, language, reasoning, mood, anxiety, depression, or motivation was recorded, along with number of months elapsed since surgery. As discussed before, any mention of a de novo impulse control disorder was recorded.

### 2.5. Statistical Analysis

Scores for each of the neuropsychological assessments were compared with published normative data. For each measure, patients were judged to be impaired if scores were ≤2 SD. When multiple measures were used, performance was classified as impaired when ≤2 SD on at least one of the measures used.

Normality of distribution was assessed using the Kolmogorov–Smirnov test and, if significant, by examining the *z*-scores for skewness and kurtosis. Homogeneity of variance was assessed using Levene's test. Unless otherwise stated, all data met the assumptions of normality and homogeneity of variance. Baseline scores of the STN and GPi DBS groups were compared using *t*-tests or Mann–Whitney tests, as appropriate. Pre- and after DBS scores were compared using *t*-tests for related samples or Wilcoxon signed-ranks, as appropriate. Pearson's correlations, chi-squared analyses, and logistic regression techniques were used to detect any significant associations. All analyses were conducted using IBM SPSS Statistics Data Editor, version 19.

The research was done in accordance with the Helsinki Declaration and the Institute of Neurology Joint Research Ethics Committee UCLH, NHS Trust Research and Development Directorate.

## 3. Results

### 3.1. Patient Demographics

As shown in Table 1, the STN and GPi DBS patient groups did not significantly differ in terms of age, gender split, or premorbid level of intellectual functioning, as estimated by the NART. They also did not significantly differ in age at diagnosis, duration of disease, or history of impulse control disorder.

### 3.2. Clinical Characteristics before and after DBS

At baseline, there were no significant differences between the STN and GPi DBS patient groups in UPDRS-III scores off or on medication, nor in baseline levodopa-equivalent dosage (as shown in Tables 2 and 3). STN DBS was successful in improving UPDRS-III scores off medication (*t*(23)=6.50, *p* < 0.001), with a corresponding reduction in levodopa-equivalent dosage (*t*(21)=4.50, *p* < 0.001). There was no significant difference in UPDRS-III scores on medication. In the GPi DBS group, there was no significant change in levodopa-equivalent dosage and change in motor performance was not examined because of insufficient collection of postsurgery motor performance data.

There were also no significant differences between the STN and GPi DBS patients groups in proportion of patients receiving dopamine agonist treatment before or after DBS.

### 3.3. Cognitive Performance before and after DBS

When baseline neuropsychological assessment scores were compared with published normative data, impairment was documented on at least one domain of cognitive function in 85% of all patients (STN: *n*=22, 64.7%; GPi: *n*=12, 100%). In both groups, impairments were frequently in intellectual functioning, memory, attention, and executive function (Table 4). The GPi DBS group also demonstrated frequent impairments in the additional domains of cognitive screen and speed. There was a significant association between DBS location and frequency of impairment, with the GPi group having more frequent impairments on the cognitive screen (*χ*^2^(1) = 9.20, *p* < 0.05), measures of memory (*χ*^2^(1) = 5.80, *p* < 0.05), executive function (*χ*^2^(1) = 9.20, *p* < 0.05), and speed (*χ*^2^(1) = 9.20, *p* < 0.05).

When investigated further, we found that the GPi DBS patients obtained lower baseline scores on tests of general intellectual functioning (VIQ: *t*(38) = 4.24, *p* < 0.001; PIQ: *t*(38) = 2.33, *p* < 0.05), recognition memory (RMT words: *U* = 65.5, *p* < 0.05; RMT faces: *t*(37) = 3.74, *p* < 0.01), attention (TEA EC with distraction: *t*(37) = 2.76, *p* < 0.05), and executive functioning (category fluency: *t*(37) = 2.75, *p* < 0.05; Stroop: *t*(35) = 3.49, *p* < 0.01; Brixton: *t*(33) = 4.12, *p* < 0.01). Thus, all subsequent analyses of cognitive performance were split according to site of DBS.

As shown in Table 5, there was a significant drop in phonemic and category fluency following both STN and GPi DBS. In the STN patients, there was also a significant decline in performance on Symbol Search, and in the GPi patients, there was also a decline in PIQ. There were also near-significant declines in Stroop performance and VIQ following STN DBS. There were no other significant or near-significant differences in cognitive performance following either STN or GPi DBS.

Case note review revealed mention of decline in cognitive function in 15% (*n*=6) of patients after DBS (4 STN DBS, 14.3%; 2 GPi DBS, 16.7%). There was no significant association between DBS location and subsequent cognitive decline (*χ*^2^(5) = 4.73, *p*=48). Number of months elapsed since surgery had a bimodal distribution, with two patients demonstrating marked decline immediately (STN and GPi DBS, resp.), but others demonstrating decline at least a year after surgery (*n*=4, range = 13–72 months). When considering those who declined immediately, one demonstrated confusion and hallucinations immediately after GPi DBS surgery, thought to be associated with a urinary tract infection and which improved with appropriate treatment consistent with a diagnosis of delirium rather than dementia. However, the other deteriorated physically and cognitively after STN DBS (as confirmed by repeat cognitive assessment), without any subsequent improvement.

### 3.4. Predictors of Cognitive Decline following DBS

Pearson correlational analysis revealed no significant baseline cognitive, mood, or motor correlates of decline in phonemic fluency after either STN or GPi DBS. Greater decline in category verbal fluency following STN DBS was associated with higher levels of apathy (*r* = 0.47, *p* < 0.05) and levodopa-equivalent dosages at baseline (*r* = −0.43, *p* < 0.05) and greater change in cognitive speed, as indexed by change in performance on both Digit Symbol Coding (*r* = 0.49, *p* < 0.05) and Symbol Search (*r* = −0.53, *p* < 0.01). However, only the correlation between decline in category fluency and Symbol Search survived the Bonferroni adjustment for multiple comparisons. Greater decline in category fluency following GPi DBS was associated with worse UPDRS-III scores off medication at baseline (*r* = 0.70, *p* < 0.05), but this did not survive the Bonferroni adjustment.

There were also no significant baseline correlates of decline in Symbol Search after STN DBS. However, greater decline in PIQ following GPi DBS was associated with slower baseline performance on the Digit Symbol Coding subtest. There were no other significant predictors of decline following DBS.

In order to identify baseline predictors of the subsequent global and irreversible cognitive decline following STN DBS noted in the one patient, Crawford and Howell [106] single-case methodology was used. This revealed that this patient was significantly older (68 years) than the mean age (59.15 years) of the STN DBS patients who remained stable (*t* = 1.86, *p* < 0.05). Indeed, although the baseline neurology assessment revealed no atypical symptoms, it did raise concerns about the older age. MMSE performance was flawless, but the patient demonstrated mild baseline impairments in all domains, including language and visuoperceptual functioning. Indeed, this patient was the only patient to demonstrate baseline impairment in language and subsequently undergo STN DBS. Another patient also demonstrated baseline impairment in visuoperceptual and subsequently underwent STN DBS, which proved successful, but it is noted that this patient was younger (55 years) than the mean age of the STN DBS group.

When considering the remaining patients who demonstrated cognitive decline at least a year after surgery (as identified in the case note review), no significant difference in demographics or cognitive performance at baseline was identified.

### 3.5. Mood before and after DBS

Baseline mood assessment revealed high rates of anxiety disorder (*n*=22, 56.4%), depression (*n*=14, 35.9%), and apathy (*n*=14, 38.9%) but no significant association between frequency of mood disorder and subsequent DBS location. There were also no significant differences in anxiety, depression, or apathy mean scores between the two surgery groups after DBS (Table 6). Case note review indicated mention of mood and/or motivation disorder in a high proportion of patients following DBS (STN: *n*=17, 60.7%; GPi: *n*=8, 66.7%), documented a mean of 23.16 months (SD = 18.09) after surgery. There was no significant association between DBS location and likelihood of mood disorder and no significant difference in time since surgery between the two DBS patient groups.

**Figure 1:**
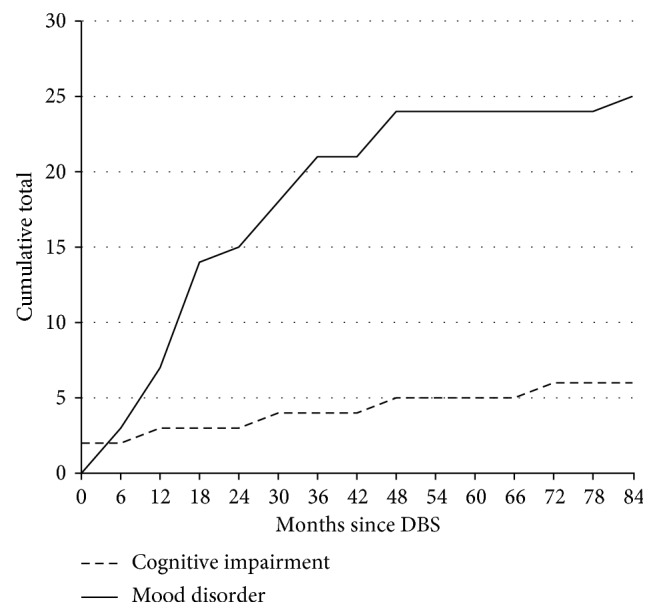
Cumulative cases of cognitive impairment and mood disorder as a function of time following DBS.

Incidence of case note indication of cognitive impairment or mood disorder, as a function of time, is depicted in Figure 1.

One patient also developed de novo impulse control disorder, namely, hypersexuality, after GPi DBS.

### 3.6. Predictors of Mood Disorder following DBS

**Figure 2:**
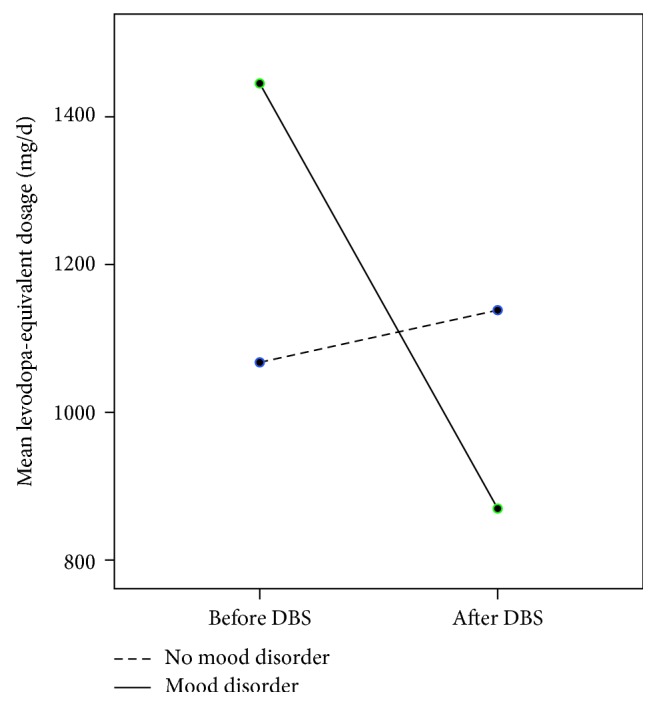
Mean levodopa-equivalent dosage of patients before and after DBS in patients split according to subsequent onset of mood disorder.

Patients who had subsequent mood disorder were found to have significantly higher baseline levels of depression (*t*(36.65) =−0 3.56, *p* < 0.01) and underwent a greater reduction in levodopa medication than those who did not (*t*(30) = −3.43, *p* < 0.01; Figure 2). There were no other significant baseline predictors of subsequent mood disorder, including DBS target.

Logistic regression confirmed these as significant predictors of subsequent mood disorder (*χ*^2^(2) = 24.13, *p* < 0.001), explaining 72.2% of the variance (Nagelkerke *R*^2^). Significant and independent associations were found for both baseline depression (*p* < 0.05; odds ratio: 2.23; 95% confidence intervals: 1.17–4.25) and levodopa reduction (*p* < 0.05; odds ratio: 1.00; 95% confidence intervals: 1.00–1.00). Classification analysis revealed only one false negative.

The patient who developed impulse control disorder following GPi DBS did not experience a reduction in levodopa-equivalent dosage but rather an increase, with ongoing dopamine agonist treatment.

## 4. Discussion

Neuropsychological assessment is considered to be an important part of the screening for selection of candidates for DBS. However, to the best of our knowledge, no standardised assessment procedure currently exists, with many studies relying upon brief screening tools only. Neuropsychological screening should comprise tests with sufficient reliability and validity, which are sensitive to cognitive impairment and dementia in PD, able to disambiguate between PD and other disorders, including atypical Parkinsonian syndromes, and be sensitive to the changes in cognitive and mood functioning associated with DBS.

In this study, we examined the use of our standardised neuropsychological assessment in a sample of patients undergoing DBS for PD. Our assessment tested a wide range of neuropsychological domains, including general intellectual functioning, verbal and visual recognition and recall memory, language, visuoperceptual functioning, attention, verbal fluency, executive functioning, and speed of processing. The tests were all standardised, with adequate psychometric properties, easy to administer, and suitable for routine clinical services.

Our neuropsychological assessment was sensitive to the cognitive impairment found in PD. At baseline, we documented frequent impairments in intellectual functioning, memory, attention, and executive function, with more frequent impairments, as expected, in the GPi group. Indeed, only six of all DBS patients (15%) did not demonstrate impairment in at least one cognitive domain. Despite this only one patient demonstrated immediate and irreversible cognitive decline following DBS. This highlights the limitation of using the presence of any baseline cognitive impairment as an exclusionary criterion for DBS. As low test scores may reflect a number of cognitive and noncognitive variables, such as high levels of fatigue, low scores on any one test should not be used to preclude surgery.

Our neuropsychological assessment was also sensitive to the cognitive impairments that warrant caution before proceeding with DBS. In the patient who demonstrated immediate and irreversible global cognitive decline, single-case statistics revealed that this patient was significantly older than the mean age of those who remained stable and had greater deficits in language and visuoperceptual processing at baseline. Of course, this is a single case, and therefore, these results may not be generalizable, but this finding supports earlier reports that decline in cognitive functioning following DBS is more common in patients who are older [12, 28, 29, 34] and who have greater or more encompassing cognitive deficits at baseline [36, 68].

Although previous guidance on patient selection has tended to focus on memory impairment as a core contraindication for surgery [45, 124], PD patients often demonstrate patchy performance on tests of memory, likely reflecting the role of frontosubcortical-mediated cognition on memory functioning [107]. In our study, we observed common impairments in memory at baseline, but frank deficits in language and visuoperceptual processing were considerably less common and likely betrayed a greater level of general cognitive impairment. Previous guidance has warned that lower cognitive functioning at baseline is predictive of poorer cognitive outcome, but hitherto, there have been no recommendations on what level of impairment should constitute a contraindication to surgery. Our data suggest that, when using the present neuropsychological assessments, caution must be advised if any deficits are revealed in language and/or visuoperceptual processing (scores <5th percentiles), particularly in patients who are older and under consideration for STN DBS.

Previous studies describing negative cognitive outcomes following DBS may have failed to identify such risk factors because of insufficient scrutiny of baseline cognitive performance. Previous reports of immediate and global decline following DBS have often stated that such deterioration has occurred despite satisfactory performance on neuropsychological testing at baseline [11, 12]). Closer examination reveals that such testing has often been limited to a few screening measures of cognitive function (e.g., the MMSE) or focused on executive function, rather than explicitly assessing the presence of impairment in others, more atypical domains, such as language and visual processing. For example, York and colleagues [12] report the immediate and global cognitive decline in one gentleman aged 73 years but limit discussion of baseline cognitive performance to MMSE only, which was notably intact with a score of 28/30.

In keeping with this, our patient who demonstrated immediate and permanent cognitive decline performed flawlessly on the MMSE and performed poorly on only two out of four tests of executive function but demonstrated unexpected impairments, most clearly in language and visual perception. This underlines the importance of a broad neuropsychological assessment, interrogating a wide range of cognitive domains, to reveal the full cognitive profile.

Our neuropsychological assessment was also sensitive to the changes in cognitive functioning associated with DBS. Pre- and after DBS assessments revealed that alongside improvements in the motor status and medication load are noted in the STN group at least, and our assessment detected significant declines in verbal fluency in both groups following DBS. This confirms the mild changes frequently noted in this cognitive function following DBS [18, 35].

Although the exact cause of verbal fluency decline remains unclear, it has been linked with reductions in self-generation [18, 22]. Accordingly, the present study found that greater decline in verbal fluency was associated with higher levels of apathy at baseline. Although this did not persist after the Bonferroni adjustment for multiple comparisons, several studies have described increases in apathy following DBS [16, 18, 22, 60, 108–110]. Such behavioural adynamia, as witnessed by the reduced fluency and increased apathy, may in part relate to changes in cognitive speed [29]. We found that reduction in fluency was significantly associated with greater changes on at least one measure of speed of processing. These changes did not seem to simply reflect withdrawal of dopaminergic medication [111, 112], as although reductions in verbal fluency were related to higher levels of baseline levodopa dosage and there was no correlation with change in dosage following DBS. It has also been suggested that the surgery itself may contribute to increases in apathy [60, 113], possibly caused by microlesions to the subthalamic area during implantation of the electrode [114].

Irrespective of the underlying mechanism, deterioration in verbal fluency can deleteriously affect activities of daily living and quality of life [115] and is correlated with reduced independence in everyday functional tasks [116]. Therefore, it is recommended that patients and their families are counselled about this significant risk before deciding to proceed with surgery, particularly those who present with higher levels of apathy at baseline.

In addition to this finding of reduced verbal fluency, our assessment detected declines in other aspects of cognitive functioning. Specifically, STN patients demonstrated significant slowing on the Symbol Search test and near-significant slowing on the Stroop and reduction in VIQ. GPi patients demonstrated significant reductions in PIQ. These findings confirm a slowing in the STN patients at least. In the absence of any other focal deficits, the heterogeneous reductions in performance on the WAIS (in both DBS groups) may also reflect the composite nature of this measure and the effortful, sustained, and speeded aspects of attentional functioning that it requires. Such reductions in speed of processing after DBS have rarely been discussed as most studies investigating cognitive changes have failed to include any measure of processing speed [115]. In previous studies, there have been conflicting reports of faster responding following STN DBS. However, further inspection suggests this may be due to a speed-accuracy trade-off [1, 117]. In our study, we have shown that deleterious changes in speed of processing are present, with likely important consequences for general intellectual functioning.

When considering the patients who went on to report cognitive decline at least a year after surgery (as identified by case note review), there were no significant predictors at baseline. This may suggest that the observed decline reflects the normal progression of the disease, rather than any preexisting vulnerability in the cognitive profile. It is important to recognise that the case note review was limited to qualitative and subjective comments only, precluding comment on the severity of any cognitive decline. However, the current findings do support previous studies which suggest that the risk of developing dementia following DBS is equivalent to that in medically treated patients [34, 118, 119]. This should be validated through future research that involves a medically treated control group.

Our study indicated no significant changes in mood or apathy, as measured by questionnaires, following DBS. However, case note review revealed a very high incidence of depression, anxiety, and/or apathy after surgery. These contrasting findings may be explained by the fact that assessment of mood relied upon self-reported symptoms of depression, anxiety, or depression, whereas case note review simply indicated clinicians' observations. Discrepancy between self- and proxy-ratings of mood in Parkinson's disease has been reported previously [45, 120, 125] and may be explained by patients' lack of insight and cognitive dysfunction [121].

Mood disorder emerging after DBS has been largely attributed to reduction in dopaminergic medications [111, 112]. Accordingly, we found that deterioration in mood was significantly correlated with reductions in levodopa medication, irrespective of DBS location. There was no association with discontinuation of dopamine agonists, suggesting that overall levodopa load was more important than the type of medication. These findings are in keeping with previous reports of mood disorder occurring as a nonmotor dopamine withdrawal syndrome after DBS [70, 112].

Furthermore, the chance of developing mood disorder, as identified in the case notes, was even higher in those who endorsed clinically significant levels of depression at baseline. This may suggest that those who have a preexisting vulnerability in mood are at high risk of developing profound mood disorder following DBS. Of course, the high incidence of mood disorder as noted in the case notes may simply reflect clinicians' recognition of (stable) low mood. However, its timing of onset and high incidence is consistent with several other studies [22, 59, 60, 122]. Therefore, we recommend careful and systematic longitudinal psychological follow-up for all PD DBS patients.

High levels of postoperative apathy or mood disorder can negate any improvement in quality of life [63, 126], but few studies have researched the presence of any baseline correlates of such decline. This study has found that a higher rating of depression at baseline is a predictor of poorer psychosocial outcome following DBS. We found high rates of depression, apathy, and anxiety in our patients at baseline, which may reflect elements of both reactive mood disorder and dysregulation of reward and motivation processing [123]. Indeed, previous research has suggested that mood disorder following DBS may reflect the effects of impaired extrastriatal dopaminergic pathways not sufficiently compensated for by STN stimulation [70]. Therefore, we would suggest that rather than excluding such patients from DBS, any dopamine withdrawal following surgery should be done cautiously.

One of our patients developed de novo impulse control disorder following GPi DBS. The onset of hypersexuality occurred in the context of increased levodopa dosages following surgery, with ongoing use of dopamine agonists. Our findings were of course limited to clinician ratings only and may have missed other cases. Future research should further investigate the incidence of impulse control disorder following DBS by using a semistructured interview, such as the QUIP [127]. Nevertheless, this case reflects the challenges of balancing treatment of motor and nonmotor symptoms in PD (cf. [128]).

### 4.1. Recommended Battery

Following our findings, we propose an abbreviated version of our neuropsychological protocol, suitable for routine clinical use. We recommend that this protocol includes our measures of current and premorbid intellectual functioning (prorated version of the WAIS-III, NART-R) to gauge overall level of intellectual decline; memory recognition and recall (RMT Words and Faces and D&P People and Shapes) to ensure cognitive profile is not amnestic and thus atypical for PD; language and visuoperceptual function (GNT and VOSP Silhouettes) to detect the identified red flags for DBS; verbal fluency (DKEFS FAS and Category) and another measure of executive function (Stroop) to determine severity of executive dysfunction; speed of processing (Digit Symbol Coding and Symbol Search); and measures of mood and behavioural functioning, targeting depression, apathy (HADS and AES), and impulse control disorder (using a measure such as the QUIP). Of course, analysis of neuropsychological performance should consider any relevant cultural or linguistic factors, and it may be appropriate to replace some of the present neuropsychological assessments with suitable substitutions for specific populations.

## 5. Conclusion

This study has presented a standardised neuropsychological assessment procedure suitable for the selection of appropriate candidates with PD for DBS and identified clear baseline risk factors for subsequent decline in cognitive functioning and mood.

## Figures and Tables

**Table 1:** Patient demographic characteristics.

	STN (*n*=28)	GPi (*n*=12)	*p*
Gender (male)	17	8	0.72
Age (at first assessment, years)	57.50 ± 7.32	61.33 ± 6.30	0.12
NART Predicted Full-Scale IQ	111.57 ± 11.08	103.42 ± 15.43	0.07
Age at PD diagnosis (years)	45.55 ± 7.80	48.60 ± 6.35	0.29
PD disease duration (years)	18.77 ± 6.12	19.00 ± 4.55	0.92
History of impulse control disorder (*n*, %)	9, 28.1%	1, 3.1%	0.08

**Table 2:** Patient motor characteristics before and after DBS.

	STN			GPi		
	Before DBS (*n*=28)	After DBS (*n*=24)	*p*	Before DBS (*n*=11)	After DBS (*n*=4)	*p*
UPDRS-III off medication	48.68 ± 14.10	28.67 ± 9.99	0.00	50.73 ± 11.09	35.20 ± 15.32	—
UPDRS-III on medication	17.29 ± 7.967	15.83 ± 7.20	0.49	24.64 ± 10.97	20.75 ± 11.56	—

**Table 3:** Patient medication characteristics before and after DBS.

	STN (*n*=28)			GPi (*n*=12)		
	Before DBS	After DBS	*p*	Before DBS	After DBS	*p*
Levodopa-equivalent dosage (mg/d)	1321.82 ± 638.68	863.73 ± 583.92	0.00	1263.40 ± 971.08	1205.10 ± 626.68	0.81
Dopamine agonist treatment (*n*, %)	15, 46.9%	6, 18.8%	0.01	7, 21.9%	5, 15.6%	0.16

**Table 4:** Cognitive performance before DBS: proportion impaired in each domain.

Cognitive domain	STN (*n*=28)	GPi (*n*=12)	*p*
Screen	**1, 3.7%**	**5, 41.7%**	**0.01**
IQ	12, 42.9%	8, 66.7%	0.30
Memory	**9, 33.3%**	**9, 75.0%**	**0.04**
Language	1, 3.7%	3, 27.3%	0.07
Perception	2, 7.4%	1, 8.3%	1.00
Attention	5, 18.5%	4, 33.3%	0.42
Executive function	**4, 14.8%**	**8, 66.7%**	**0.01**
Speed	**2, 7.4%**	**4, 36.4%**	**0.05**

Results are given as number and percentage. Chi-squared significant group comparisons are indicated in bold.

**Table 5:** Cognitive performance before and after DBS: mean scores on each test.

Assessment	STN (*n*=28)			GPi (*n*=12)		
	Before DBS	After DBS	*p*	Before DBS	After DBS	*p*
MMSE (30)	28.64 ± 1.41	28.64 ± 1.68	1.00^a^	26.75 ± 3.14	25.75 ± 2.87	0.31^a^
WAIS-VIQ	111.21 ± 12.40	107.54 ± 15.93	0.05^a^	92.75 ± 13.10	92.67 ± 10.71	0.97^a^
Vocabulary (66)	51.57 ± 9.61	49.96 ± 10.00	0.13^a^	40.64 ± 15.02	37.27 ± 14.14	0.16^a^
Similarities (33)	**24.71 ± 4.51**	**22.75 ± 5.64**	0.02^a^	19.36 ± 5.43	18.36 ± 5.12	0.43^a^
Arithmetic (22)	**15.61 ± 2.94**	**14.04 ± 4.64**	0.01^a^	10.36 ± 2.94	9.64 ± 3.04	0.90^a^
Digit span (30)	**17.57 ± 4.15**	**16.43 ± 4.26**	0.04^a^	14.08 ± 3.09	14.00 ± 3.16	0.41^a^
WAIS-PIQ	106.37 ± 15.17	104.04 ± 19.57	0.50^a^	**93.64 ± 15.11**	**84.45 ± 12.91**	**0.01** ^**a**^
Picture Completion (25)	17.96 ± 4.25	17.19 ± 4.86	0.33^a^	12.92 ± 3.66	11.33 ± 3.53	0.07^a^
Matrix Reasoning (26)	16.35 ± 5.61	15.31 ± 5.90	0.24^a^	11.10 ± 4.33	9.20 ± 4.21	0.09^a^
RMT-W (50)	46.81 ± 3.50	45.12 ± 5.35	0.10^b^	39.20 ± 9.45	40.30 ± 8.68	0.16^a^
RMT-F (50)	41.88 ± 4.13	41.08 ± 5.68	0.54^a^	33.60 ± 6.85	34.00 ± 7.92	0.80^a^
D&P People delayed (12)	7.21 ± 3.68	7.50 ± 4.30	0.59^b^	6.40 ± 4.65	5.60 ± 3.95	0.57^a^
D&P Shapes delayed (12)	10.50 ± 3.28	10.25 ± 2.82	0.22^b^	8.86 ± 3.63	7.43 ± 3.78	0.30^a^
GNT (30)	23.69 ± 3.42	23.69 ± 3.28	1.00^a^	17.91 ± 8.11	19.45 ± 6.65	0.34^a^
VOSP Silhouettes (30)	22.81 ± 3.25	21.92 ± 3.91	0.13^a^	20.82 ± 3.52	19.00 ± 5.88	0.41^a^
DKEFS FAS (SS)	**13.42 ± 4.89**	**11.54 ± 4.61**	**0.01** ^**a**^	**10.92 ± 5.18**	**8.00 ± 4.88**	**0.01** ^**a**^
DKEFS Category (SS)	**12.31 ± 4.21**	**10.00 ± 4.99**	**0.01** ^**a**^	**8.50 ± 3.00**	**5.00 ± 3.30**	**0.01** ^**a**^
Stroop (112)	91.81 ± 21.36	83.77 ± 22.94	0.06^a^	63.00 ± 20.44	58.50 ± 23.45	0.12^a^
Hayling (SS)	5.68 ± 1.07	5.32 ± 1.52	0.28^b^	4.60 ± 1.84	4.70 ± 1.83	0.89^a^
Brixton (SS)	4.91 ± 1.53	5.00 ± 2.28	0.83^a^	2.33 ± 1.66	2.56 ± 2.07	0.72^a^
TEA EC (7)	6.67 ± 0.96	6.75 ± 0.44	0.85^b^	6.50 ± 1.41	5.88 ± 1.55	0.26^b^
TEA EC-Distraction (SS)	9.91 ± 2.66	8.96 ± 2.92	0.15^a^	7.13 ± 3.14	5.75 ± 1.91	0.17^a^
WAIS-SS (SS)	**9.62 ± 2.25**	**8.46 ± 2.82**	**0.02** ^**a**^	7.89 ± 3.33	6.11 ± 2.42	0.86^a^
WAIS-DSC (SS)	8.20 ± 2.52	7.48 ± 2.65	0.22	4.89 ± 2.67	4.67 ± 1.87	0.86^a^

Results are given as mean ± SD (^a^paired *t*-test; ^b^Wilcoxon signed-rank). Significant differences are indicated in bold. MMSE: Mini-Mental Status Examination; WAIS-VIQ, PIQ: Wechsler Adult Intelligence Scale-Third Edition-Verbal IQ, Performance IQ; RMT-W, F: Warrington Recognition Memory Test for Words, Faces; D&P: Doors and People Test; GNT: Graded Naming Test; VOSP: Visual Object and Space Perception Battery; DKEFS: Delis–Kaplan Executive Function System; SS: scaled score; TEA EC, ECD: Test of Everyday Attention Elevator Counting, Elevator Counting with Distraction; WAIS-III SC, DSC: Wechsler Adult Intelligence Scale-Third Edition Symbol Search, Digit Symbol Coding.

**Table 6:** Mood scores before and after DBS.

Assessment	STN (*n*=28)			GPi (*n*=12)		
	Before DBS	After DBS	*p*	Before DBS	After DBS	*p*
HADS anxiety (21)	7.50 ± 3.23	6.27 ± 4.85	0.19^b^	8.55 ± 3.30	9.00 ± 4.12	0.69^a^
HADS depression (21)	6.15 ± 4.42	6.00 ± 4.04	0.86^a^	7.00 ± 3.85	7.73 ± 4.63	0.67^a^
Apathy (54)	10.75 ± 6.02	13.96 ± 11.16	0.15^b^	14.57 ± 6.71	20.86 ± 11.11	0.67^a^

Results are given as mean ± SD (^a^paired *t*-test; ^b^Wilcoxon signed-rank).
